# Genetic interaction between *TTG2* and *AtPLC1* reveals a role for phosphoinositide signaling in a co-regulated suite of *Arabidopsis* epidermal pathways

**DOI:** 10.1038/s41598-024-60530-8

**Published:** 2024-04-28

**Authors:** Aleah Goldberg, Patrick O’Connor, Cassandra Gonzalez, Mason Ouren, Luis Rivera, Noor Radde, Michael Nguyen, Felipe Ponce-Herrera, Alan Lloyd, Antonio Gonzalez

**Affiliations:** 1 Department of Molecular Biosciences and The Institute for Cellular and Molecular Biology, The University of Texas at Austin, 2500 Speedway, Austin, TX 78712 USA; 2 The Freshman Research Initiative, The University of Texas at Austin, Austin, TX 78712 USA

**Keywords:** *TTG2*, *AtPLC1*, Phosphoinositides, Lipid signaling, Trichomes, Seed coat, Proanthocyanidins, MBW, Cell biology, Genetics, Plant sciences

## Abstract

The TTG2 transcription factor of *Arabidopsis* regulates a set of epidermal traits, including the differentiation of leaf trichomes, flavonoid pigment production in cells of the inner testa (or seed coat) layer and mucilage production in specialized cells of the outer testa layer. Despite the fact that TTG2 has been known for over twenty years as an important regulator of multiple developmental pathways, little has been discovered about the downstream mechanisms by which TTG2 co-regulates these epidermal features. In this study, we present evidence of phosphoinositide lipid signaling as a mechanism for the regulation of TTG2-dependent epidermal pathways. Overexpression of the *AtPLC1* gene rescues the trichome and seed coat phenotypes of the *ttg2-1* mutant plant. Moreover, in the case of seed coat color rescue, *AtPLC1* overexpression restored expression of the TTG2 flavonoid pathway target genes, *TT12* and *TT13/AHA10*. Consistent with these observations, a dominant *AtPLC1* T-DNA insertion allele (*plc1-1D)* promotes trichome development in both wild-type and *ttg2-3* plants. Also, *AtPLC1* promoter:GUS analysis shows expression in trichomes and this expression appears dependent on TTG2. Taken together, the discovery of a genetic interaction between *TTG2* and *AtPLC1* suggests a role for phosphoinositide signaling in the regulation of trichome development, flavonoid pigment biosynthesis and the differentiation of mucilage-producing cells of the seed coat. This finding provides new avenues for future research at the intersection of the TTG2-dependent developmental pathways and the numerous molecular and cellular phenomena influenced by phospholipid signaling.

## Introduction

A suite of co-regulated epidermal traits in *Arabidopsis* has served as one of the preeminent systems for studying the genetics of cell differentiation, patterning and organ formation^1–3^. These epidermal developmental pathways lead to trichome (hair) initiation and patterning, the differentiation of the mucilage-producing outer testa (seed coat) layer, the production of flavonoid-based proanthocyanidin (PA) pigments (also known as condensed tannins) in the inner seed coat layer, and root hair patterning. Extensive genetic studies of these plant epidermal features identified the combinatorial MYB-bHLH-WDR (MBW) transcriptional complex regulatory model. The *transparent testa glabra 1* (*ttg1*) mutant of *Arabidopsis* defined a key member of the MBW complex and highlighted the co-regulation of seemingly disparate epidermal traits^4,5^. *TTG1* encodes a small WD-repeat containing protein, which serves as a platform for protein–protein interactions. TTG1 physically interacts with combinations of R2R3 MYB and bHLH transcription factors to specify specific epidermal cell fates^1–3^

An additional layer of transcriptional control of the TTG1-dependent epidermal pathways exists just downstream of the MBW complex^6–11^. Two transcription factors, Glabra2 (GL2), a homeodomain Leu-zipper (HD-ZIP) protein, and Transparent Testa Glabra2 (TTG2), a WRKY protein, are themselves major direct transcriptional targets of the MBW complex. GL2 and TTG2 regulate events in all of the MBW-dependent developmental programs, representing key control nodes in these programs. Both GL2 and TTG2 have positive roles in trichome initiation and development, and in seed coat mucilage production. GL2 also regulates root hair patterning while TTG2 functions in PA biosynthesis in the seed coat.

*TTG2* was originally identified as a trichome and seed coat mutant from a transposon tagging screen^8^. The *ttg2-1* loss of function mutant shows fewer and underdeveloped, mostly unbranched trichomes compared to wild-type, suggesting a primary role in trichome outgrowth and branch initiation. The seed coats of *ttg2-1* mutants fail to differentiate properly; the inner testa layer lacks PA pigments while the outer layer cells do not differentiate to produce mucilage. *TTG2* also contributes to maternal control of seed size by regulating integument cell elongation^12^. Consistent with these pleiotropic phenotypes, *TTG2* is expressed in trichomes and developing seed coats^8^. *TTG2* is also expressed in non-hair root epidermal cell files, but no mutant root hair patterning defect is obvious in *ttg2-1* mutants. Additionally, *TTG2* is expressed in the endosperm throughout seed development where it appears to have a role in preventing endosperm cellularization^13^.

*TTG2* encodes a 429 amino acid protein containing two WRKY plant transcriptional regulation domains. The WRKY domain is a DNA binding domain of about 60 amino acids containing the conserved WRKY motif along with a novel zinc finger motif^14–16^. WRKY proteins show high affinity for the W box defined as (T)(T)TGAC(T/C). As a family, WRKY genes tend to mediate biotic and abiotic stress responses, so TTG2 is notable in that it functions in development as well.

Some of the molecular and biochemical mechanisms of TTG2 regulation and its relationship to the MBW complex have been uncovered. In the trichome pathway, TTG2 directly regulates the *Triptychon* (*TRY*) gene, encoding a R3 MYB negative regulator of trichome initiation^17,18^. In the flavonoid pigment pathway, TTG2 regulates PA precursor vacuolar transport steps encoded by *Transparent Testa 12* (*TT12*) and *AHA10*/*Transparent Testa 13* (*TT13*)^19^. Also, TTG2 physically interacts with TTG1, and TTG2 requires TTG1 for target gene activation in the trichome and PA pathways^17,19^.

Several lines of evidence, by way of gene expression analysis, suggest a possible role for PLC signaling in trichome development. Microarray experiments showed that *AtPLC1* (At5G58670) is expressed about 24-fold higher in trichome cells compared to the non-trichome cells of the leaves from which they were harvested^20^. Promoter:GUS reporter analysis showed that *AtPLC2* is expressed in trichomes^21^, although no trichome phenotypes in *atplc2* mutant plants were reported. Also *AtPLC7* promoter:GUS analysis shows expression in trichomes^22^. Promoter:GUS analysis has also revealed that *AtPLC3* is expressed in support cells at the base of trichomes. Moreover, phospholipid signaling was originally implicated years ago in at least one of the MBW-regulated pathways by the discovery that root hair patterning requires the *AtPLDζ1* gene which is a direct target of GL2^23^.

To further study potential roles of phospholipid signaling in the *TTG2-dependant* epidermal traits, we focused our analysis on the *AtPLC1* gene (At5G58670). We found that *AtPLC1* is expressed in trichomes and this expression is reduced in trichomes of *ttg2-1* mutants. Moreover, overexpression of *AtPLC1* rescues the phenotypes of the *ttg2-1* mutant. In the case of the seed coat PA pigment pathway, overexpression of *AtPLC1* in *ttg2-1* plants restored the expression of *TT12* and *AHA10/TT13*. In addition, an *AtPLC1* T-DNA insertion mutant line, *plc1-1D* (S025769C), shows increased trichome initiation and branching, and this phenotype is inherited dominantly. Also, the *plc1-1D* allele rescues the trichome phenotype of *ttg2-3* mutants. Curiously, overexpression of *AtPLC1* in L*er* wild-type seedlings inconsistently results in underdeveloped trichomes. Taken together, these observations point to a novel TTG2 regulatory mechanism involving phospholipid signaling for the coordinate control of trichome and seed coat development.

## Results

### *AtPLC1* is expressed in trichomes and this expression is dependent on TTG2

**Figure 1:**
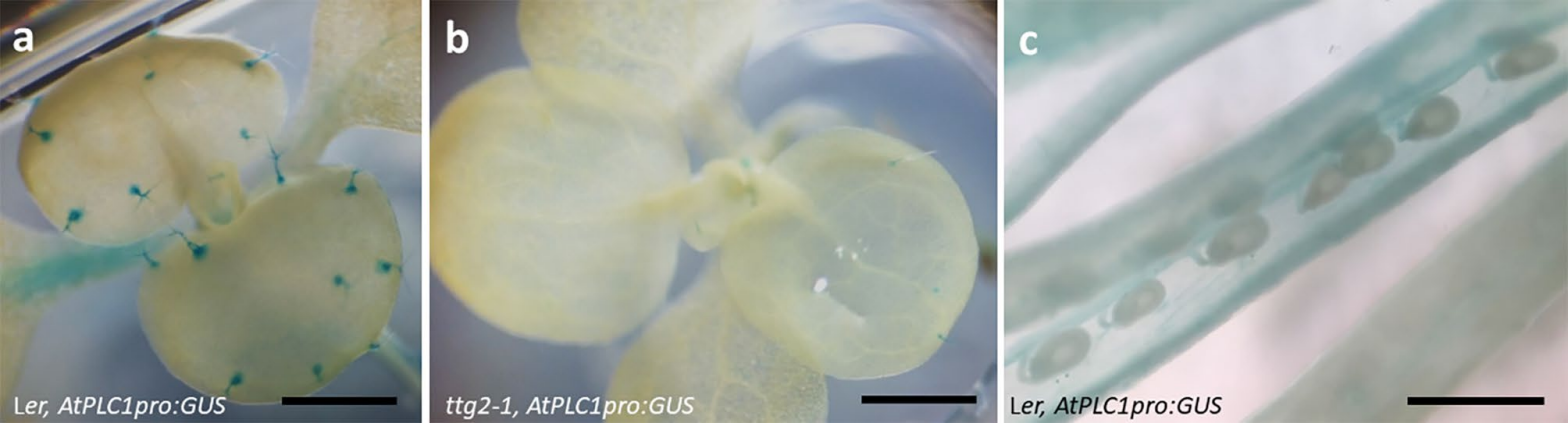
*AtPLC1pro:GUS* expression pattern in seedlings and developing seed. (**a**) L*er* seedling with expanded first true leaves and emerging third and fourth leaves. (**b**) *ttg2-1* seedling with expanded first true leaves and emerging third and fourth leaves. (**c**) Dissected L*er* silique showing developing seed at approximately the late heart stage. Scale bars: 500 µm.

To confirm trichome specific expression of the *AtPLC1* gene, we created promoter:GUS reporter constructs for *AtPLC1*. We found that L*er* ecotype reporter lines harboring the *AtPLC1pro:GUS* construct showed trichome specific expression on the leaves of young seedlings (Fig. 1a). This expression is reduced in the trichomes of *ttg2-1* reporter liners (Fig. 1b).

Because trichome and seed coat development are co-regulated by *TTG2*, we checked the expression of *AtPLC1* in developing seed. *AtPLC1pro:GUS* reporter L*er* lines showed no obvious expression in developing seed coats (Fig. 1c).

### Overexpression of *AtPLC1* in Ler suppresses trichome development

**Figure 2:**
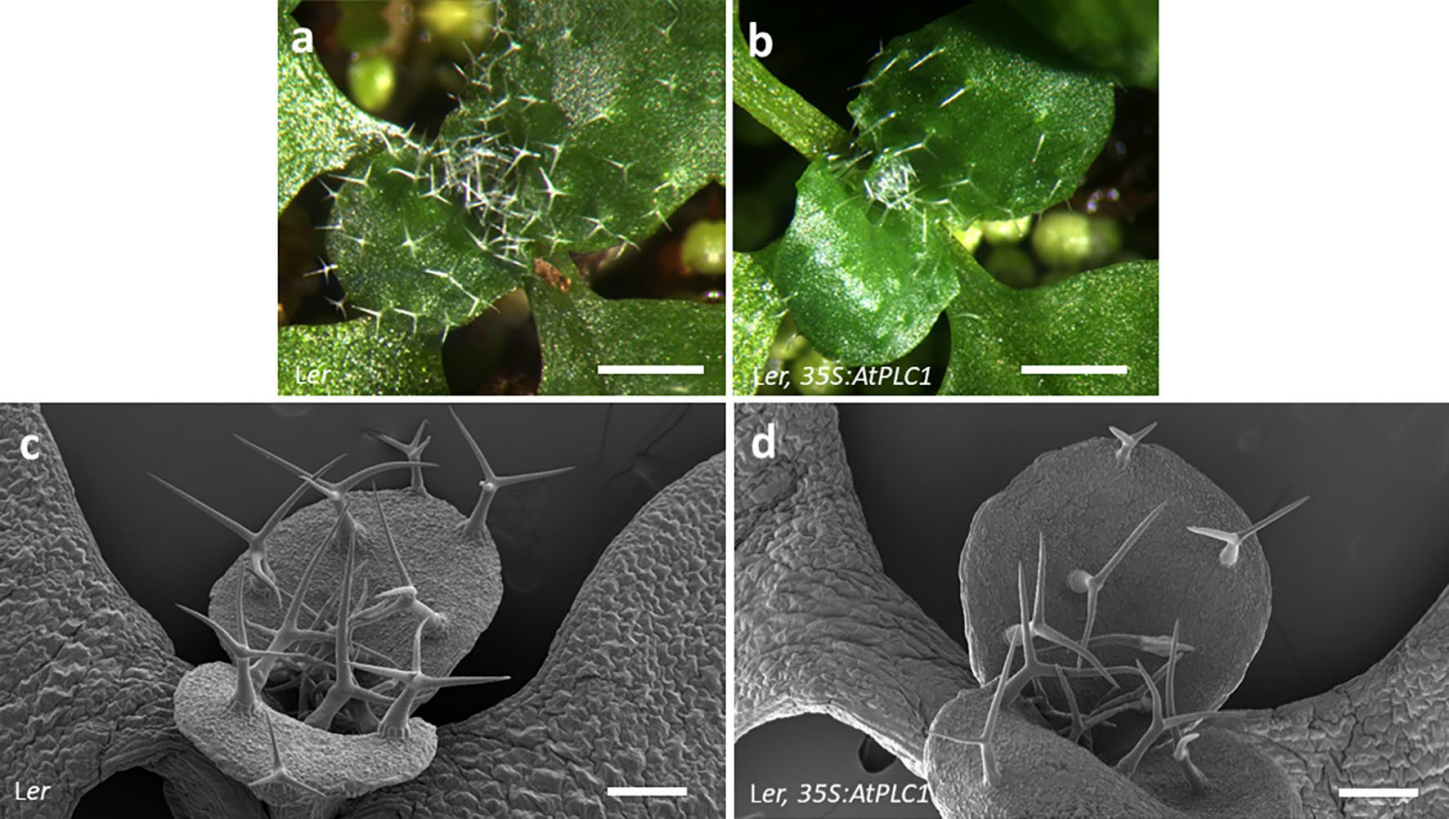
*AtPLC1* overexpression in L*er* seedlings. Light micrographs of expanding third and fourth leaves on (**a**) L*er* seedling and (**b**) *35S:AtPLC1* L*er* transgenic seedling. (**c**) and (**d**) SEM of expanding first true leaves on L*er* seedling and *35S:AtPLC1* L*er* transgenic seedling, respectively. Scale bars: a and b = 500 µm, c and d = 100 µm.

To investigate possible roles of *AtPLC1* in *Arabidopsis*, we made constructs expressing the *AtPLC1* gene under the control of the constitutive cauliflower mosaic virus 35S promoter (*35S:AtPLC1*). The *35S:AtPLC1* construct in L*er* resulted in underdeveloped trichomes in some of the primary transgenic lines (Fig. 2). Instead of the three-branched trichomes typically found on L*er* seedlings, the trichomes of *35S:AtPLC1* L*er* seedlings were primarily two-branched (Fig. 2c,d). No obvious seed coat phenotypes were observed in seeds of *35S:AtPLC1* plants.

### Overexpression of *AtPLC1* in *ttg2-1* plants rescues mutant trichome and seed coat phenotypes

**Figure 3:**
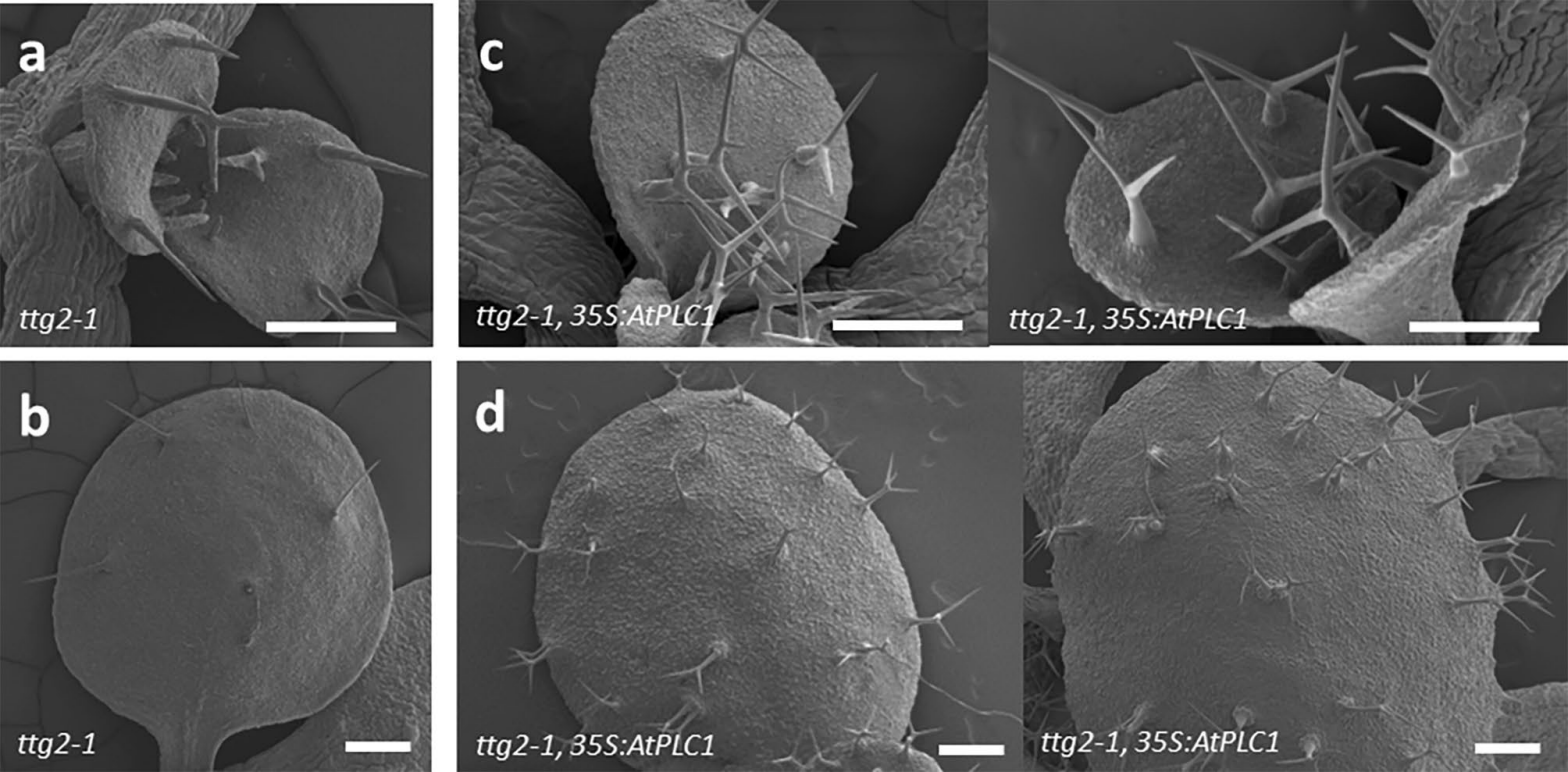
SEMs of *AtPLC1* overexpression in *ttg2-1* seedlings. (**a**) *ttg2-1* seedling with expanding first true leaves and (**b**) An expanded *ttg2-1* first true leaf. (**c**) Two *35S:AtPLC1 ttg2-1* transgenic seedlings with expanding first true leaves and (**d**) Two expanded first true leaves from *35S:AtPLC1 ttg2-1* transgenic seedlings. Images are from a single transgenic line representative of other lines. Scale bars: 200 µm.

**Figure 4:**
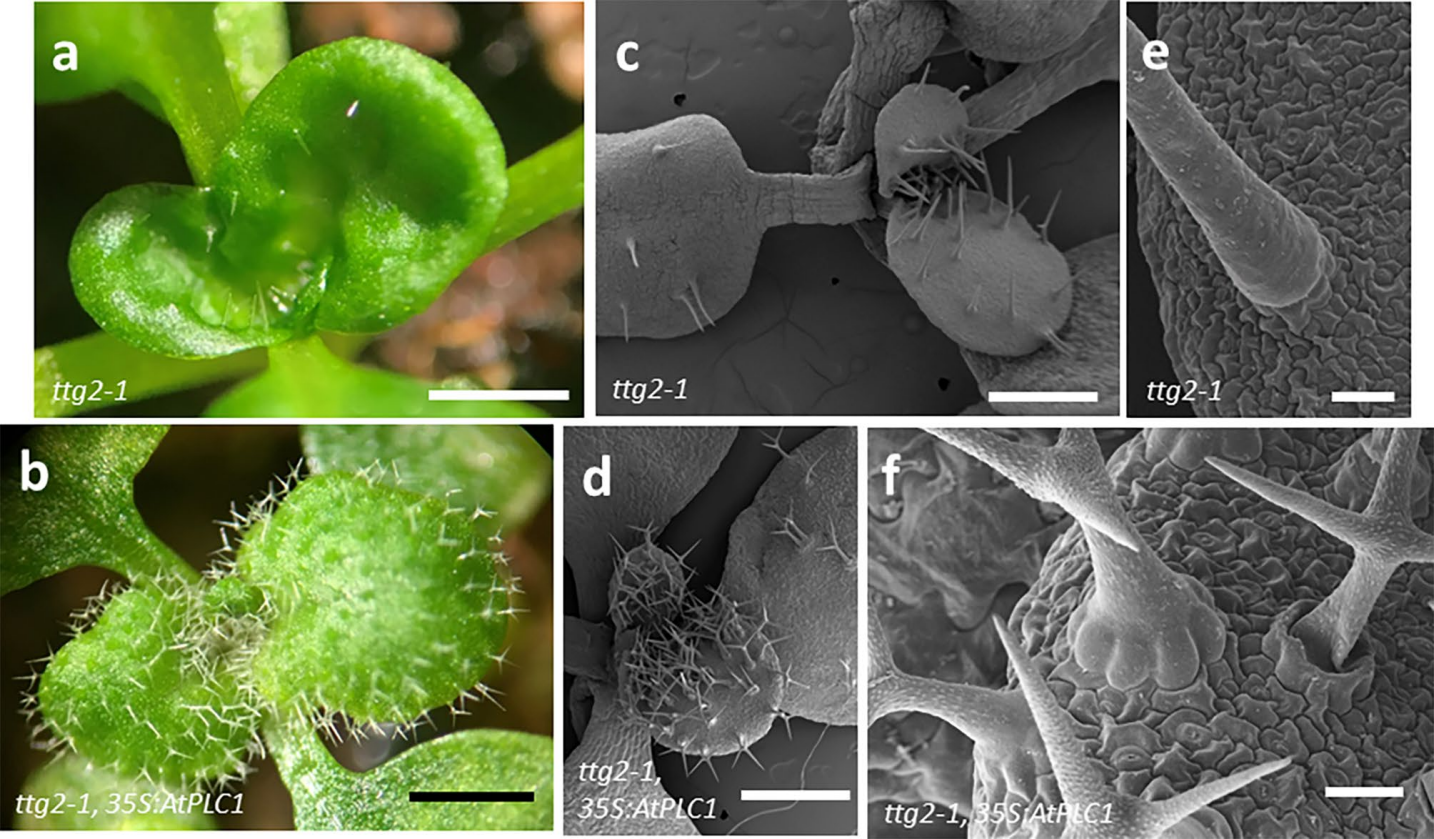
*AtPLC1* overexpression in third and fourth leaves *ttg2-1* seedlings. Light micrographs of expanding third and fourth leaves on (**a**) *ttg2-1* seedling and (**b**) *35S:AtPLC1 ttg2-1* transgenic seedling. (**c**) *ttg2-1* seedling with expanding third and fourth leaves. (**d**) A *35S:AtPLC1 ttg2-1* seedling with expanding third and fourth leaves. (**e**) Trichome on *ttg2-1* mutant seedlings and (**f**) trichomes on *35S:AtPLC1 ttg2-1* seedling. Scale bars: a-d = 500 µm, e = 20 µm, f = 50 µm.

Surprisingly, *ttg2-1* plants overexpressing *AtPLC1* showed rescued trichome development. The trichomes on first true leaves of *ttg2-1* seedlings are typically unbranched (Fig. 3a,b), but trichomes on *ttg2-1* seedlings overexpressing *AtPLC1* were two and three-branched (Fig. 3c,d). On the third and fourth leaves (Fig. 4), trichome initiation, as well as branching, was increased in the overexpressor compared to untransformed *ttg2-1* plants. In addition, some trichome clustering (in pairs) was observed on leaves of *35S:AtPLC1 ttg2-1* plants (Fig. 3d), indicating a disruption in trichome patterning (clustering of trichomes is not observed in wild-type plants). Lastly, trichomes on the overexpressor lines appeared to be terminally differentiated, as evidenced by a loss of the glassy appearance under light microscopy and increased or enhanced papillae on the cell surface as imaged under SEM (Fig. 4a,b,e and f).

**Figure 5:**
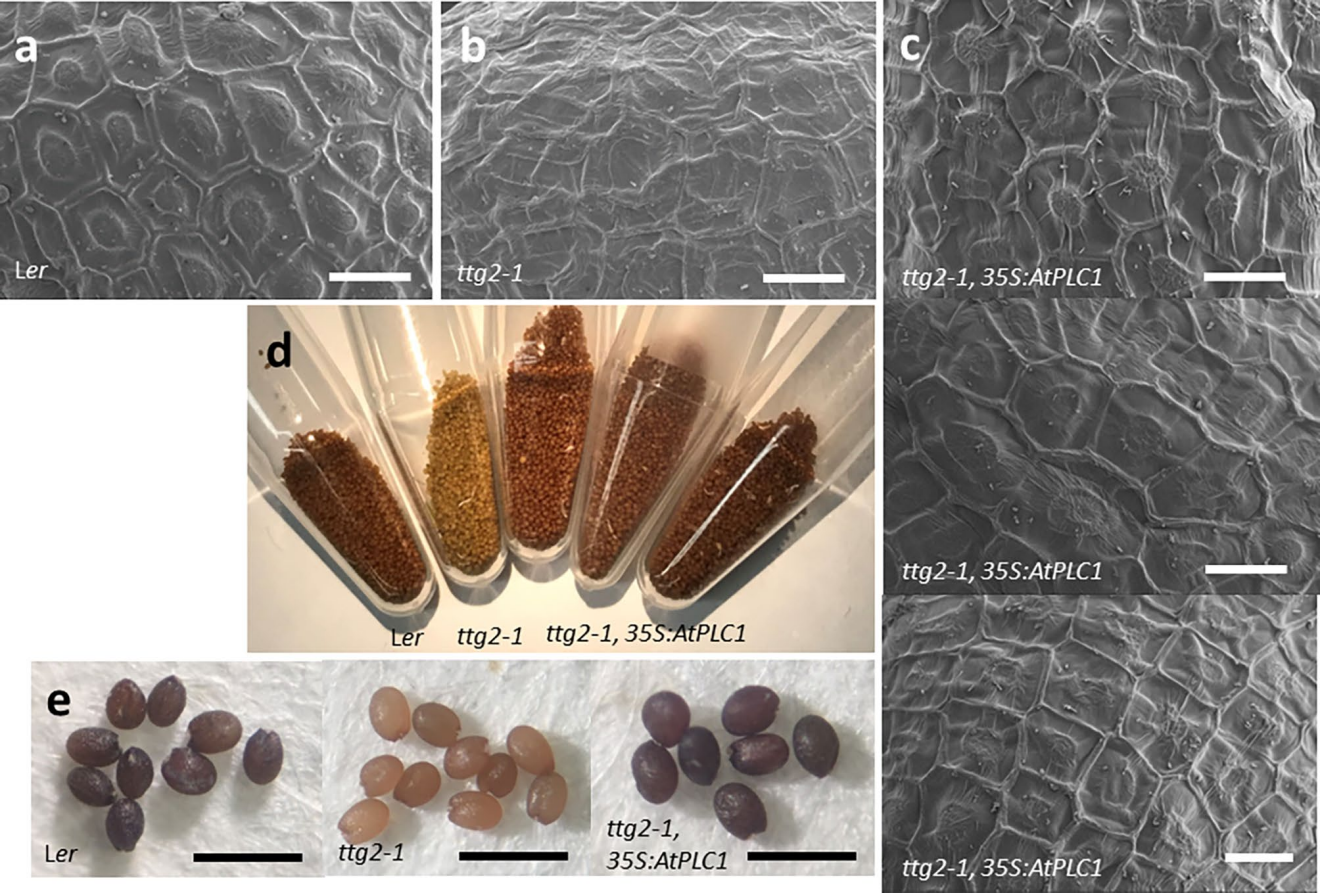
*AtPLC1* overexpression in *ttg2-1* seeds. SEM of (**a**) *Ler* and (**b**) *ttg2-1* dry seed surface. (**c**) three representative images of dry seed surface showing the mucilage-producing outer testa cells of *35S:AtPLC1 ttg2-1* seed. (**d**) Dry seed color phenotypes from left to right: L*er*, *ttg2-1* and seed from three lines of *35S:AtPLC1 ttg2-1* plants. (**e**) DMACA stained dry seed from L*er*, *ttg2-1*, and *35S:AtPLC1 ttg2-1* plants. Scale bars: a-c = 20 µm, e = 1 mm.

Seed development was also rescued in *ttg2-1* plants overexpressing *AtPLC1.* Under SEM, the cells of the outer testa layer of wild-type dry seeds showed the characteristic appearance of raised columellae surrounded by reinforced radial walls^11,24^ (Fig. 5a). These cells on *ttg2-1* seed showed no columella formation and indistinct radial walls (Fig. 5b). However, seeds from *ttg2-1* plants overexpressing *AtPLC1* showed a range of partially to mostly rescued cell morphologies (Fig. 5c).

In addition, seed color appeared to be mostly restored in *ttg2-1* plants overexpressing *AtPLC1* (Fig. 5d). DMACA reagent was also used to stain PAs and epicatechin pigment precursors in L*er*, *ttg2-1* and *35S:AtPLC1 ttg2-1* dry seed^25,26^. DMACA staining further showed restoration of PA and pigment precursor production in *35S:AtPLC1 ttg2-1* lines (Fig. 5e). *ttg2-1* mutant seeds showed very low DMACA staining but seed from L*er* and *35S:AtPLC1 ttg2-1* lines showed positive, dark DMACA staining.

### Overexpression of *AtPLC1* in *ttg2-1* mutant restores the expression of *TT12* and *AHA10/TT13* PA pathway genes

**Figure 6:**
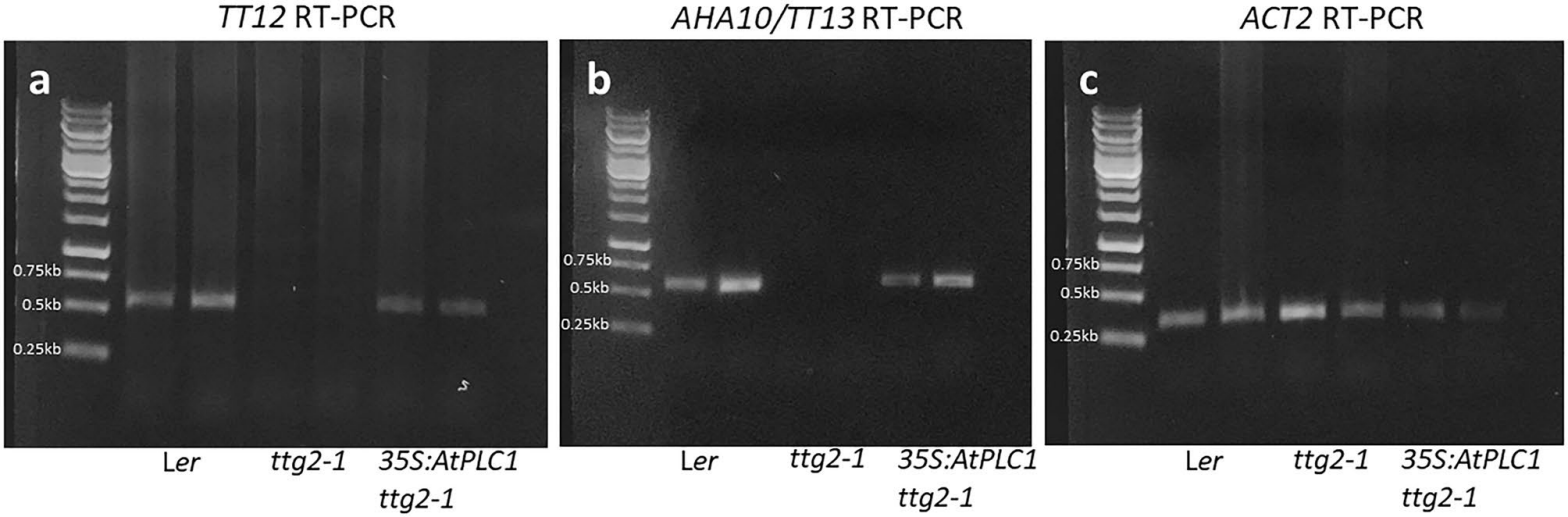
Semi-quantitative RT-PCR gene expression of *TT12* and *AHA10/TT13* in L*er*, *ttg2-1* and *35S:AtPLC1 ttg2-1*. Amplification of (**a**) *TT12*, (**b**) *AHA10/TT13* and (**c**) *ACT2* amplicons from two biological replicates of L*er*, *ttg2-1*, and *35S:AtPLC1 ttg2-1* first-strand cDNAs. Images (a, b and c) are from three independently run gels.

Because *AtPLC1* overexpression rescued PA pigment and pigment precursor production in *ttg2-1* seeds, we employed semi-quantitative RT-PCR to check for *TT12* and *AHA10/TT13* expression in developing siliques. As previously reported^19^, *TT12* and *AHA10/TT13* expression was detected in L*er* developing silique, but not detected in *ttg2-1* developing silique (Fig. 6a and b). However, *TT12* and *AHA10/TT13* expression was detected in RT-PCR reactions containing first strand cDNA prepared from silique of *ttg2-1* plants overexpressing *AtPLC1* (Fig. 6a and b). Expression of the actin control was detected in all RT-PCR reactions (6c).

### An *AtPLC1 T-DNA* insertion line, *plc1-1D*, shows increased trichome initiation and branching phenotypes that are inherited dominantly, and rescues the *ttg2-3* trichome phenotype

We observed a discrepancy regarding the phenotypic results of overexpressing *AtPLC1* in L*er* plants (Fig. 2) vs. *ttg2-1* mutants (Figs. 2 and 3). To help resolve the phenotypic discrepancy between L*er* and *ttg2-1* lines overexpressing *AtPLC1*, we acquired an *AtPLC1* T-DNA homozygous insertion line (*plc1-1D*) in the Col-0 accession (Salk 025769C) from the *Arabidopsis* Biological Resource Center^27^ (Alonso et al.; 2003). In this line, the insertion is immediately after the 126th base pair (or 42nd codon) in the first exon^22^ (Kanehara et al., 2015). The *plc1-1D* insertion line showed an increase in trichome branching, with the four-branch class increasing to 61% from 13% observed in Col (Table 1). Moreover, five and six-branched trichomes, which are not observed in Col first true leaves, were observed on first true leaves of the T-DNA insertion line: 20% of trichomes in the *plc1-1D* line were five-branched while 2% were six-branched. Also, the *plc1-1D* line showed increased trichome initiation, with first true leaves containing about 41% more trichomes than Col first true leaves (Table 1). When Col plants were crossed to the *plc1-1D* line, the F1 progeny showed increased trichome branching and an increase in initiation, indicating that the *plc1-1D* phenotype is inherited dominantly, and that the insertion allele is likely a gain-of-function mutation (Table 1).

**Table 1:** Trichome numbers and branching in wild-type and *plc1-1D* insertion line.

genotype	Branching phenotype			Average trichome number		Total
	3 (%)	4 (%)	5 (%)	6 (%)		
Col	87	13	0	0	21.3 (2.1)	383
*plc1-1D*	17	61	20	2	30.1 (6.1)	487
Col x *plc1-1D*, F1	31	66	3	0	27.3 (3.6)	273

Trichomes on pairs of expanded first true leaves were counted and the branch phenotype (3, 4, 5 and 6 branches) is reported as the percentage of trichomes with the indicated phenotype. Average trichome number is for a first true leaf for each of the indicated plant lines, with the standard deviation in parentheses. Total is the total number of trichomes counted on first true leaves of seedlings of each line. Trichomes on first true leaf pairs on approximately 18, 16 and 10 seedlings were counted for Col, *plc1-1D* and Col *x plc1-1D* F1 lines, respectively.

**Figure 7:**
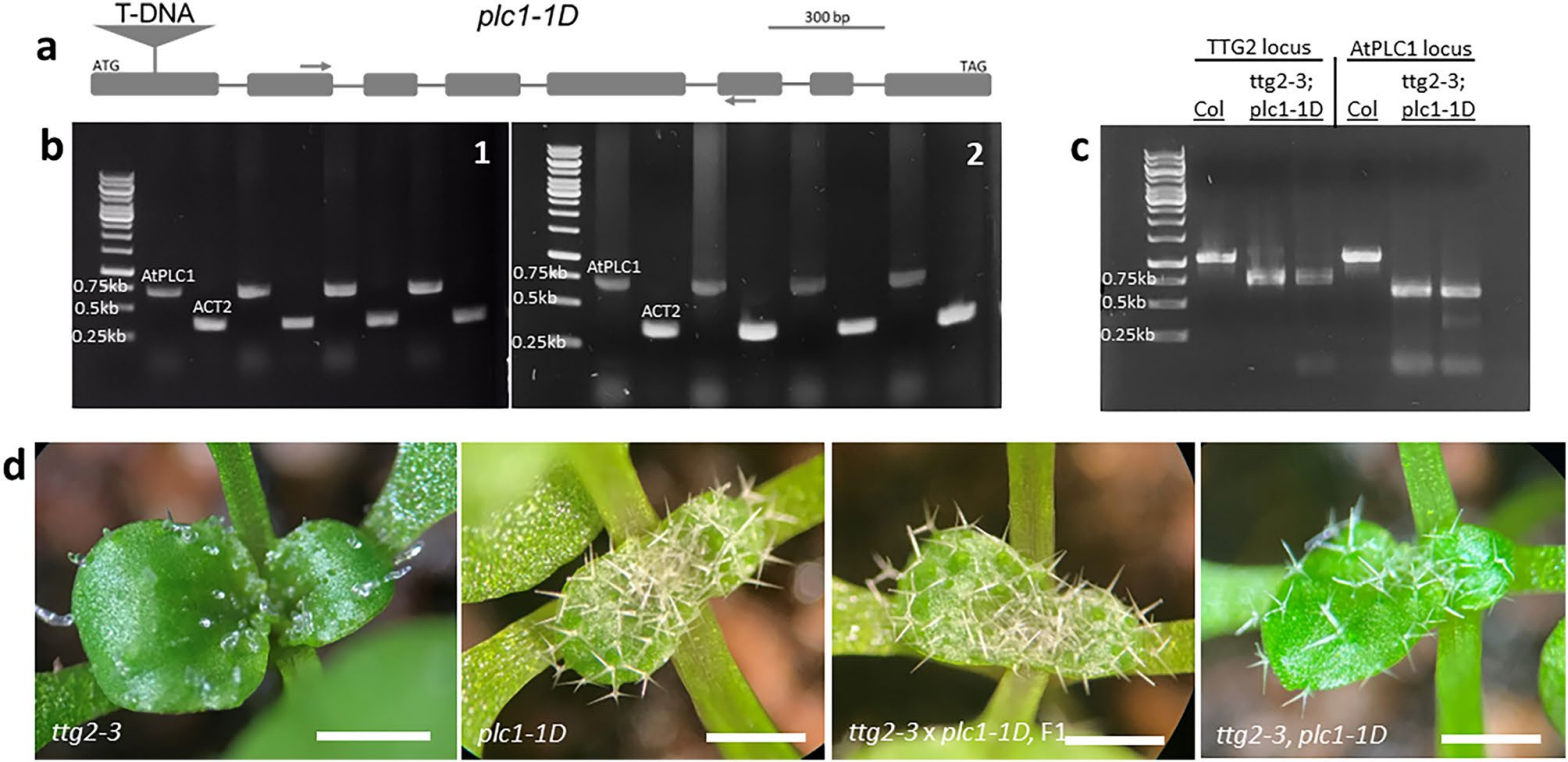
Characterization of the *AtPLC1* T-DNA mutant, *plc1-1D*. (**a**) Schematic model of the *AtPLC1* gene. Shown are the general locations of T-DNA insertion site in exon 1 and the primers downstream of then insertion for detecting *AtPLC1* expression in RT-PCR. Gray boxes depict exons, lines depict introns, the triangle depicts the insertion and arrows depict the RT-PCR primers. A scale bar for the exons of approximately 300 bp is shown. (**b**) Semi-quantitative RT-PCR gene expression of *AtPLC1* in Col and *plc1-1D* mutant: 1. Amplification of *AtPLC1* (larger band) and *ACT2* (smaller band) from four biological replicates of Col first-strand seedling cDNA, 2. Amplification of *AtPLC1* (larger band) and *ACT2* (smaller band) from four biological replicates of *plc1-1D* mutant first-strand seedling cDNA. (**c**) Genotyping results for *ttg2-3; plc1-1D* double mutant. Lane 1 shows the DNA ladder. Lanes 2–4 shows *TTG2* locus genotyping in Col-0 and in two double mutant candidates; lanes 5–7 show *AtPLC1* locus genotyping in Col-0 and in two double mutant candidates. For both genes tested, larger band signifies wild-type alleles and smaller bands signify TDNA insertion alleles. (**d**) Trichome phenotypes on emerging third and fourth true leaves. From left to right: *ttg2-3*; *plc1-1D* mutant; F1 individual from a cross between *ttg2-3* and *plc1-1D* mutants*; ttg2-3; plc1-1D* double mutant. Scale bars: d = 500 µm.

To investigate possible mechanisms by which the *plc1-1D* allele might remain functional, we conducted semi-quantitative RT-PCR to check for any expression from the *AtPLC1* locus downstream of the insertion. We used a primer set 354 bp downstream of the T-DNA insertion to amplify a 727 bp fragment of *AtPLC1* from first-strand seedling cDNA. Also, the primers spanned four introns of the *AtPLC1* genomic locus such that amplification from genomic DNA would result in a 1042 bp amplicon (Fig. 7a). As expected, *AtPLC1* expression was detected in Col-0 seedling cDNA (Fig. 7b). *AtPLC1* expression was also detected in cDNA prepared form the *plc1-1D* mutant line, as evidenced by the amplification of the 727 bp RT-PCR fragment (Fig. 7b). This indicates that the *AtPLC1* locus in the T-DNA mutant is not transcriptionally silent, leaving the possibility that the remaining 519 codons after the insertion may yield some truncated protein with potential functionality. Expression of the actin control was detected in all RT-PCR reactions.

To further characterize the genetic nature of the *plc1-1D* line, we crossed the *ttg2-3* T-DNA insertion mutant in the Col-0 accession (Salk 148838C) to the *plc1-1D* line. The *ttg2-3* loss-of-function mutant has been previously described^13^. The trichome phenotype of *ttg2-3* mutant plants was noted as showing few and aberrant trichomes. Seeds of *ttg2-3* plants also lack PA pigment like *ttg2-1* seed^13^. We note that *ttg2-3* trichomes showed a range of mutant phenotypes including swollen, glassy, abnormally branched, and generally distorted trichomes (Fig. 7d).

The F1 of a cross between *ttg2-3* and the *plc1-1D* lines showed normal looking, branched, terminally differentiated trichomes (Fig. 7d). The F1 was allowed to self and the F2 seedlings showed two qualitatively distinct trichome phenotypes: (1) the aberrant *ttg2-3* parental trichome phenotype and (2) normal looking, differentiated, branched trichomes. Out of 263 F2 seedlings scored, 17 showed the *ttg2-3* trichome phenotype and 246 seedlings showed the developed, terminally differentiated trichome phenotype. This phenotypic ratio is 1:14.5, close to the 1:15 F2 phenotypic ratio that would be expected if the *plc1-1D* allele is dominant and rescues the *ttg2-3* trichome phenotype. A chi-square test of the F2 data results in a critical value of 0.02, indicating that the small deviation from the predicted 1:15 phenotypic ratio is about 90% probable on the basis of chance. Because we expect 4/16 of the F2 population to be of the genotype *ttg2-3/ttg2-3* but only observe the *ttg2-3* trichome phenotype at a frequency of about 1/15, this suggests that one copy of the *plc1-1D* allele is sufficient to rescue the trichome phenotype of *ttg2-3* homozygous mutants.

To further demonstrate the rescue of *ttg2-3* phenotypes by the *plc1-1D* allele, we isolated plants with the genotype *ttg2-3/ttg2-3; plc1-1D/plc1-1D* (Fig. 7c and d)*.* These plants show wild-type looking trichomes despite being homozygous for the *ttg2-3* mutant allele. Overall, these results are consistent with a gain-of-function mutation in the *plc1-1D* line that rescues the *tt2-3* mutant. This result is also consistent with the observation that *AtPLC1* overexpression rescues the *ttg2-1* mutant.

## Discussion

In *Arabidopsis*, multiple epidermal traits including trichomes, flavonoid pigment production in the shoot and inner seed coat layer, mucilage production in the outer seed coat layer, and root epidermal cell patterning, are co-regulated by MBW transcription factor complexes^1–3^. This regulatory circuit includes two key downstream transcription factor targets, *TTG2* and *GL2*. The regulation of this suite of epidermal traits radiates from these two control nodes, with *TTG2* and *GL2* controlling an overlapping set of the TTG1-dependent epidermal pathway.

The discovery of *AtPLDζ1* as a GL2 target over 20 years ago represents a fascinating but lone example of phospholipid signaling as part of the MBW-GL2 regulatory circuit controlling the development of the root epidermis in *Arabidopsis*^23^. AtPLDζ1 presumably cleaves phosphatidylcholine into choline and a phosphatidic acid. Phosphatidic acid has emerged as a key signaling lipid in plants, regulating an array of cellular processes including vesicle trafficking and actin cytoskeleton organization^28^. In the context of root epidermal cell fate, it might be that vesicle trafficking and/or translocation of proteins to the growing root hair tip necessary for cell morphogenesis is regulated by AtPLDζ1^23^.

In this study, we introduce a link between the epidermal traits co-regulated by the MBW-TTG2 regulatory circuit and phosphoinositide lipid signaling, specifically involving a phospholipase C gene, *AtPLC1*. Implicating PLC signaling in the TTG2-dependent suit of epidermal pathways should provide fertile ground for future research into plant phosphoinositide regulation of molecular, cellular and developmental processes.

### *AtPLC1* overexpression effects MBW-TTG2 epidermal traits but with some discrepant results

Transgenic plants overexpressing *AtPLC1* show changes in trichome phenotype in both the L*er* wild-type and *ttg2-1* mutant lines. However, L*er* transgenic lines show reduced trichome development while *ttg2-1* transgenic lines show substantial degrees of suppression of the mutant trichome phenotype. In addition, *AtPLC1* overexpression rescues *ttg2-1* mutant seed coat mucilage production and seed coat color, including the restoration of PA pathway structural gene expression in *ttg2-1* developing siliques. However, no obvious reduction in seed coat development was detected in L*er* plants overexpressing *AtPLC1* (data not shown).

These initial results beg the questions: does *AtPLC1* promote or inhibit TTG2-dependent epidermal pathways? Why the contradictory trichome phenotypes in wild-type plants and *ttg2-1* mutants overexpressing *AtPLC1?* To begin with the latter question, it is possible the opposing trichome phenotype results are due to a difference in the genetic backgrounds of the wild-type and the mutant lines, and their interplay with regulatory circuitry having both positive and negative control mechanisms. The *ttg2-1* mutant might represent a sensitized genetic background in which the promoting effects of *AtPLC1* are revealed, while the wild-type genetic background is primed to reveal inhibition of the trichome pathway. In fact, such a phenomenon has been observed, in which the R2R3 MYB GL1 activates both trichome initiation and inhibition so that trichomes normally differentiate in a pattern as isolated cells rather than in clusters. Loss-of-function *gl1* mutants are bald due to a lack of initiation of the trichome cell fate^29^. However, when *GL1* is overexpressed in wild-type plants, trichome initiation is largely suppressed, due to over-activation of R3 MYB repressors that interfere with functional MBW complexes^30,31^. When *GL1* is overexpressed in mutant plants lacking the TRY R3 MYB repressor of trichome initiation, only then is an overproduction of trichome cells observed^32^. Indeed, similar to GL1, TTG2 promotes trichome initiation and development, while also directly regulating the trichome inhibitor gene, *TRY*^17^. Thus, the downregulation of TRY is a potentially consequential genetic difference between L*er* and *ttg2-1* plants overexpressing *AtPLC1*. It will be interesting to see in future studies the effects of overexpressing *AtPLC1* in other trichome genetic backgrounds such as R3 MYB repressor mutants as well as initiation mutants.

Regardless, this study shows that *AtPLC1* can influence multiple MBW-TTG2 dependent epidermal traits; *AtPLC1* overexpression not only affects trichomes, but also rescues seed coat development in *ttg2-1* mutants. Moreover, expression of the TTG2 PA pigment pathway gene targets, *TT12* and *AHA10/TT13*, is restored in *ttg2-1* plants overexpressing *AtPLC1*. Taken together, these data suggest that *AtPLC1* positively mediates *TTG2* control of this co-regulated group of epidermal traits.

### A T-DNA insertion line sheds more light on the biological role of *AtPLC1* in TTG2-dependent epidermal pathways

A search for an *AtPLC1* knockout line among the Salk T-DNA insertion collection ironically yielded a dominant mutant allele that resulted in enhanced trichome phenotypes. Nonetheless, this insertion mutant, *plc1-1D* (Salk 025769C), provided more insights to the role of this lipid signaling gene in epidermal development. When crossed to the Col wild-type accession, all the F1 progeny showed a phenotype intermediate between both parents, representing an enhancement of trichome development compared to the Col parent (Table 1). In addition, the *plc1-1D* allele appears to rescue the trichome phenotype of the *ttg2-3* mutant, given the 15:1 wild-type to mutant phenotypic ratio among the F2 progeny of a cross between *ttg2-3* and *plc1-1D* lines. Also, plants homozygous for both *ttg2-3* and *AtPLC1* insertion alleles show normal-looking trichomes (Fig. 7). This result is reminiscent of *AtPLC1* ectopic expression rescuing the *ttg2-1* mutant and again suggests that *plc1-1D* allele is a gain-of-function.

Lastly, these crossing data help resolve the observation that *AtPLC1* overexpression in wild-type plants reduces trichome development but rescues in *ttg2* mutant plants. The dominant *plc1-1D* allele acts as a positive regulator of trichome development in both wild-type and *ttg2* plants. Overall, the varied genetic data using the dominant *plc1-1D* line in wild-type backcrosses and dihybrid crosses with *ttg2-3,* the *35S:AtPLC1* data in *ttg2-1*, and the *AtPLC1pro:GUS* data in L*er* and *ttg2-1* are consistent with the hypothesis that *AtPLC1* genetically interacts with *TTG2* to positively regulate multiple epidermal developmental pathways. As previously discussed, the MBW-TTG2 regulatory circuit both promotes and inhibits epidermal development^17,19^. Thus, the trichome pathway is sensitive to perturbations that disrupt the stochastic balance of co-regulated activating and inhibiting elements of the pathway. It is therefore possible that overexpression of *AtPLC1* via the 35S promoter represents a strong perturbation of the system towards inhibition, while the T-DNA insertion allele represents a more subtle, localized boost in function, and thus a smaller perturbation towards promoting the trichome pathway, leading to different outcomes. Similar to previous studies of *GL1*, these various observations using the *35S:AtPLC1* construct, and the *plc1-1D* allele in wild-type plants and sensitized *ttg2* mutant lines, might again be revealing a dual capability for genes that regulate MBW-controlled epidermal pathways. Future work will focus on examining more closely the possible positive and negative regulatory roles of *AtPLC1* in TTG2-dependent epidermal pathways.

### The phosphoinositide signaling pathway regulates various cellular and biological functions in plants consistent with putative roles in the MBW-TTG2 controlled epidermal traits

Phosphoinositides (PIs) are a class of negatively charged glycerophospholipids found in eukaryotic membranes. PIs are derived from the phosphorylation of phosphatidylinositol (PtdIns), a family of lipids characterized by the attached myo-inositol head group. The myo-inositol head group can be phosphorylated at the 3, 4 and 5 positions of the inositol ring to yield phospotidylinositol-monophosphates (PIP) and bisphosphates (PIP_2_). Relative to other membrane lipids, PIs are present in very small quantities that are in a dynamic state of flux. This constant turnover is due to the action of kinases, phosphatases and lipases comprising the PI pathway (for excellent reviews on the PI pathway and its roles in plant biology, see references^33–38^).

PIs and their signaling derivatives exert profound effects. They regulate multiple facets of cell biology such as actin cytoskeleton organization, cell wall synthesis, vacuole morphology and function, vesicle trafficking, and nuclear functions^33,36–38^. Accordingly, perturbations to the levels of PIs and their signaling products via manipulation of PI pathway genes can result in severe phenotypes. This phenotypic approach has yielded much insight to the biological roles of this important lipid-signaling pathway^38^.

Indeed, previous genetic studies of the PI pathway held clues indicating possible roles for lipid metabolism genes in at least some of the epidermal traits regulated by *TTG2*. For example, the *fragile fiber7* (*fra7*) mutant gene was found to be allelic to *AtSAC1*, encoding a PI phosphatase exhibiting in vitro activity towards PI(3,5)P_2_^39^. Fiber and pith cells of the *fra7* mutant show aberrant cell morphogenesis, cell wall synthesis and actin organization. Interestingly, trichomes of the *fra7* mutant when imaged by scanning electron microscopy appeared stunted and lacked surface papillae, indicating a lack of secondary cell wall thickening, a characteristic feature of terminal trichome differentiation.

Additionally, *AtIPK1* encodes an inositol pentakisphosphate (IP_5_) kinase that produces inositol hexakisphosphate (IP_6_). Both IP5 and IP6 are important signaling molecules ultimately derived by phosphorylation of the inositol head group cleaved from the phosphoinositides PI4P and PI(4,5)P_2_ by PLCs. Plants harboring the *atipk1-1* mutant allele show reduced growth and a long root hair phenotype associated with defects in phosphate sensing and signaling^40^. *AtIPK1* promoter:GUS analysis showed expression in several cell types and tissues, including trichomes and developing seeds, possibly suggesting a function for this gene in some of the same cells and tissues co-regulated by *TTG2*.

Interestingly, IP5 is a cofactor for the F-box protein Coronatine Insensitive 1 (COI1), which mediates the jasmonic acid (JA) wounding response. The *atipk1* mutant also shows increased sensitivity to JA (presumably due to the increase in IP5), and thus an enhanced wounding response and a more robust defense against insect herbivory^36,41^. Part of the herbivory-induced wound response mediated by JA signaling is activation of the trichome and anthocyanin MBW transcriptional complexes, subsequently resulting in increased trichome initiation and anthocyanin production^42,43^. This represents another possible connection between PI signaling and the epidermal pathways co-regulated by the MBW-TTG2 transcriptional network via a positive feedback loop reinforcing the wound response: JA mediated activation of the MBW complex would in turn activate *TTG2* gene expression, possibly resulting in increased PLC signaling, yielding the IP5 cofactor of COI1.

The phosphoinositide bisphosphate PI(4,5)P2 and the enzymes such as PLCs that metabolize this PI are known to have roles in several biological processes. These range from pollen tube and root hair growth, hormone signaling, stomatal opening/closure, developmental phase shifts, and biotic and abiotic stress response^34,35^. Also, PLC signaling has been shown to regulate female gametogenesis and embryo development, and plant immunity in *Arabidopsis*^44,45^. Lastly, it’s been shown that *plc5 plc7* double mutants are deficient for seed coat mucilage production^22^, a trait regulated by *TTG2*. Here, we show that genetic perturbations targeting the activity of the *AtPLC1* gene affect the *TTG2* co-regulated suite of epidermal traits, revealing new biological roles for PLCs and the PI pathway in plant development. However, the exact mechanisms by which PLC signaling influences these epidermal pathways remain unclear, thus providing opportunities for future work.

### Possible modes of PLC-mediated PI(4,5)P_2_ signaling in the regulation of plant cellular functions

**Figure 8:**
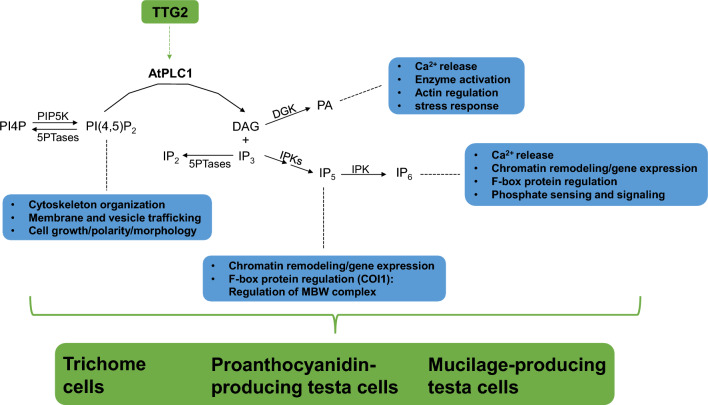
Model for the integration of PLC-mediated phosphoinositide lipid signaling in the regulation of the TTG2-dependent epidermal cell fate pathways. Green boxes indicate the TTG2 transcription factor and the co-regulated epidermal traits. In between is the PLC lipid signaling pathway with blue boxes indicating the cellular functions influenced by the various branches and signaling metabolites of the pathway. The regulation of such cellular functions are likely important for the development of trichomes and cells of the seed coat. PLC lipid signaling is therefore a possible mechanism for the regulation of cellular functions by which TTG2 controls epidermal development. The genetic interactions between *TTG2* and *AtPLC1* observed in this study suggests such a mechanism. This model provides a framework for further exploring the possible roles phosphoinositide lipid signaling in the TTG2-dependent epidermal cell fate pathways.

The canonical model for PLC signaling to emerge from animal studies involves extracellular receptor-activated PLCs cleaving PI(4,5)P_2_ into inositol 1,4,5-trisphosphate (IP_3_) and diacylglycerol (DAG)^46^ (Fig. 8). IP_3_ is free to diffuse through the cytosol where it activates ligand-gated channels, releasing stored Ca^2+^ into the cytosol. This Ca^2+^ concentration increase in the cytosol results in signal transduction effecting many downstream components of the signaling cascade. Similarly, DAG, the lipid cleavage product, diffuses through the membrane to activate protein kinase C (PKC), with subsequent phosphorylation of downstream signal transducers prompting a variety of cellular responses.

Although early studies in plants showed that IP_3_ could release intracellular stores of Ca^2+^ into the cytosol, resulting in stomatal closure, no obvious homolog for the IP_3_-gated Ca^2+^ channel has been identified in plant genomes to date. Likewise, identification of plant genes homologous to animal PKC genes have not been forthcoming^33,35,47^. Instead, phosphorylated versions of both IP_3_ and DAG second messengers appear to be the key downstream elicitors of the cellular responses to plant PLC activity (Fig. 8). For example, IP_6_ can trigger Ca^2+^ release from intracellular stores, and IP_5_ and IP_6_ bind to auxin and jasmonate hormone receptors, respectively, ultimately resulting in the regulation of gene expression. Likewise, the PLC lipid cleavage product DAG can be phosphorylated to form phosphatidic acid, which has emerged as an important second messenger in plants^28^.

Alternatively, PLCs may be operating to effect signaling by a different mechanism. PI(4,5)P_2_ may not merely be a precursor of second messengers, but can itself as an intact phospholipid be a mediator of cellular functions. The negatively charged head group of PI(4,5)P_2_ can serve as a ligand for a diverse array of proteins containing one of a number of lipid binding domains^36^. For example, actin-binding proteins can bind to PI(4,5)P_2_, thus regulating actin cytoskeleton organization.

Also, organization of PI(4,5)P_2_ pools to specific locations on the plasma membrane, or microdomains, is a key mechanism by which PI(4,5)P_2_ exerts its cellular functions^33,38^. For example, in growing root hairs and pollen tubes, PI(4,5)P_2_ is localized to the apical end of the cell, establishing the necessary polarity that directs the secretory pathway to the growing end^48–51^. Accordingly, the enzymes that synthesize and/or degrade PI(4,5)P_2_ may be localized to the sub-apical region as required to maintain the appropriate lipid microdomain for normal root hair or pollen tube growth. Disruption of this apical phospholipid microdomain by an increase or decrease of the enzymes that metabolize PI(4,5)P_2_ results in growth defects and abnormal morphologies of root hairs and pollen tubes.

According to the observations presented here, the differentiation of trichomes and seed coat cells also seemingly depend on PI(4,5)P_2_. Either PI(4,5)P_2_ is serving as a precursor for downstream second messengers produced by PLC activity, or as an intact phospholipid whose turnover rate or membrane localization influences the cellular processes necessary for trichome and seed coat cell development, or both (Fig. 8). These observations contribute to previous studies establishing the importance of PI(4,5)P_2_ metabolism for normal plant cell growth. The model systems co-regulated by the MBW-TTG2 regulatory circuit provide a new venue to genetically dissect the PI signaling pathway. Identifying other PI pathway genes expressed in these epidermal cell types could expand the list of candidates with roles in these cell fate pathways. In addition, regulating PI(4,5)P_2_ levels via targeted expression of PI pathway genes in these model systems could yield instructive phenotypes, providing insights to the modes of PI(4,5)P_2_ action and expanding upon the biological roles of the PI signaling pathway^33,38^.

### PLC-mediated PI pathway lipid signaling can regulate gene expression in the proanthocyanidin pathway

It was interesting to discover that overexpression of *AtPLC1* restored *TT12* and *AHA10/TT13* gene expression in the *ttg2-1* mutant (Fig. 5d and e). To our knowledge, there has not been a direct link between phospholipid signaling and proanthocyanidin pigment production in plants. The control of *AtPLDζ1* by *GL2* may suggest an indirect link between anthocyanin biosynthesis and phospholipid signaling. *GL2* has been shown to negatively regulate anthocyanin biosynthesis in *Arabidopsis* seedlings and stems^52^. Given that *GL2*-mediated root hair patterning requires the AtPLDζ1 gene^23^, it is possible that phospholipid signaling also has a role in *GL2* regulation of the anthocyanin pathway (although this has not been empirically demonstrated).

Thus, the observation that *AtPLC1* restored pigment gene expression in the *ttg2-1* mutant highlights that the regulation of gene expression is at least part of the molecular mechanisms by which *AtPLC1* might be influencing TTG2-dependent epidermal traits. Phosphoinositide and inositol polyphosphate messengers in plants and animals are known to influence nuclear functions including transcription and mRNA export^40,46,53,54^. In the context of this nuclear pathway, these lipid-derived messengers and the enzymes catalyzing their synthesis and degradation are active in the nucleus, where they influence chromatin modification.

For example, the *Arabidopsis* trithorax factor (ATX1), a histone H3 trimethylase, modifies the nucleosomes of the WRKY70 gene, positively influencing its transcription. However, upon dehydration stress, PI5P levels increase due to the activity of the PI(3,5)P2 phosphatase AtMTM1. PI5P then binds to ATX1, altering its localization from the nucleus to the cytoplasm. This results in decreased methylation of the nucleosomes of *WRKY70* and downregulation of its expression^53^.

In another example, mutations in the *AtIPK1* gene leads to a decrease of IP_6_ levels, which correlate to altered histone composition in chromatin and ultimately increases the expression of phosphate starvation response genes^40^. In yeast and animal systems, PI(4,5)P2 is present within the nucleus, along with a number of enzymes involved in PI(4,5)P2 signaling, including PLCs^54,55^. From within the nucleus, PI(4,5)P2 signaling can influence a broad range of phenomena including chromatin structure, transcription, telomere function and mRNA processing and export. In the context of AtPLC1 and PI lipid regulation of the PA pigment pathway, it will be interesting to further investigate the nuclear functions, such as chromatin remodeling/histone modifications, that might be influencing the transcription of *TT12* and *AHA10/TT13*.

### Future work: a genetics approach targeting the misexpression of other PI enzymes could clarify modes of phosphoinositide signaling action in the regulation of the MBW-TTG2 system of epidermal pathways

A phenotypic approach upon perturbation of the PI signaling pathway has proven fruitful for identifying the relevance of phospholipids as regulators of a wide range of plant biology^38^. In this study, such an approach has revealed that phospholipid signaling, particularly mediated by PLC activity, can influence multiple TTG2-dependent epidermal developmental pathways. Future genetic studies will focus on identifying the possible roles for other PI pathway genes in this system of co-regulated epidermal traits, particularly (but not limited to) those genes involved in PI(4,5)P2 metabolism (Fig. 8). These studies should also have the potential to discriminate between signaling modes of action. For example, if increased AtPLC1 signaling in *35S:AtPLC1-ttg2-1* transgenic lines is occurring via the generation of inositol polyphosphates, then co-expressing phosphatases targeting these second messengers might counteract PLC signaling, resulting in a reversal of mutant phenotype rescue. Such an approach overexpressing a type I inositol polyphosphate 5-phosphatase (5PTase) encoded by the *AtIP5PII* gene, and targeting IP_3_, reversed AtPLC1-mediated seed dormancy and plant growth inhibition in the abscisic acid signaling pathway^56,57^.

Alternatively, if *ttg2-1* mutant phenotype rescue due to *AtPLC1* overexpression is a function of decreased PI(4,5)P_2_ levels (and not the generation of second messengers), then expressing phosphatases thought to target PI(4,5)P_2_, such as *Suppressor of Actin 9* (*SAC9*) or *SAC7*^50^, might similarly result in *ttg2-1* mutant phenotype rescue. Similarly, a type II 5PTase encoded by *Fragile Fiber 3* influences actin organization and cell wall thickening in fiber and xylem cells by targeting PI(4,5)P_2_^58^, providing another means to genetically manipulate PLC signaling by reducing PI(4,5)P_2_ levels in the context of TTG2-dependent epidermal pathways.

Also, several PI4P 5-kinases that synthesize PI(4,5)P_2_ from PI4P are known to effect cellular functions such as vacuole morphology, directional vesicle trafficking, membrane recycling, localized pectin deposition, and ultimately regulate cell growth polarity and morphology of pollen tubes and root hairs^48,51,59–62^. If expressing these kinases in *35S:AtPLC1-ttg2-1* plants reverses the phenotype rescue, then this similarly would validate the hypothesis that levels of intact PI(4,5)P_2_ are important for regulating cell fate determination in the TTG2-dependent epidermal pathways. Beyond PI(4,5)P_2_ and PLC activity, it will be interesting to investigate other PI pathway enzymes and phospholipids known to regulate cellular functions important for the differentiation of trichomes and testa cells (such as actin organization, vesicle trafficking, cell wall synthesis and vacuole biology regulated by a variety of phosphoinositides and their metabolic enzymes).

## Materials and methods

### Arabidopsis accessions and transgenic lines

The mutant line *ttg2-1* is in the Landsberg *erecta* (L*er*) ecotype and has been previously described^4,5,8^. The *plc1-1D* (Salk 025769C) and *ttg2-3* (Salk 148,838) insertion mutant lines in Col-0 background were obtained from the *Arabidopsis* Biological Resource Center^27^. Four *35S:AtPLC1* lines were generated in the *ttg2-1* mutant background and two in the L*er* background, and these transgenics were phenotypically characterized. Data documented in this study are representative of common trends observed in the transgenic lines. All plants were grown in soil at approximately 21 °C in continuous white light.

### Microscopy

Scanning electron microscopy was performed as previously described^24,63^.

### Plasmid construction

pAtPLC1pro:GUS- an approximately 2 kb fragment upstream of the *AtPLC1* start codon was amplified from Col-0 genomic DNA using the primers below and recombined into pDONR222 (Invitrogen) to produce pEAtPLC1pro. pEAtPLC1pro was then used to recombine the *AtPLC1* regulatory fragment into pBGWFS7 GUS vector^64^. Gateway recombination sequences were included on all appropriate primers but are not shown.

AtPLC1profwd: 5′attB1- CAGGAGCGATTCCTTTACTAG-3′.

AtPLC1prorev: 5′attB2- CTTGTGAAAGTTAAGCGAG-3′.

p35S:AtPLC1- the *AtPLC1* Col-0 genomic locus from start to stop codons was amplified using the primers below and recombined into pDONR222 (Invitrogen) to produce pEAtPLC1. *AtPLC1* was then recombined from pEAtPLC1 into pB7WG2^64^.

AtPLC1fwd: 5′attB1- ATGAAAGAATCATTCAAAGTG-3′.

AtPLC1rev: 5′attB2- CTAACGAGGCTCCAAGACAAA-3′.

### TT12 and AHA10 gene expression analysis by semi-quantitative RT-PCR

Total RNA was prepared from young developing siliques, about 2–5 daf or during the globular stage, using a Qiagen RNeasy plant mini kit. 2 μg of total RNA was used to produce first-strand cDNA in 20 μl reverse transcription reactions using a Super-Script III RT kit (Invitrogen). 50 μl PCR reactions were prepared using 1 μl cDNA reaction as template and run for 25 cycles. 5ul of completed PCR reactions were checked on 1% agarose gels. Target primers were used at 200 nM final concentration and 200 nM actin primers were used in separate control reactions. Two biological replicates were performed for each experiment. The following primers were used for target and control gene amplification:

AHA10 fwd: 5′-TGCCAACAACAGTGAACAAGTG-3′.

AHA10 rev: 5′-TTAGACAGTATGAGCTGCACGG-3′.

TT12 fwd: 5′-GGGATATGCAGTTCATGCTTGG-3′.

TT12 rev: 5′-TTAAACACCTGCGTTAGCCATC-3′.

ACT2 fwd: 5′-AGAAGTCTTGTTCCAGCCCTC-3′.

ACT2 rev: 5′-TTAGAAACATTTTCTGTGAACG-3′.

### *AtPLC1* Gene expression analysis by semi-quantitative RT-PCR

Total RNA was prepared from young seedlings, with expanded 1st leaves and emerging 3rd and 4th leaves, using a Qiagen RNeasy plant mini kit. 3 μg of total RNA was used to produce first-strand cDNA in 20 μl reverse transcription reactions using a Super-Script III RT kit (Invitrogen). For AtPLC1 fragment amplification, 50 μl PCR reactions were prepared using 5 μl cDNA reaction as template and run for 30 cycles. For the actin control, 50 μl PCR reactions were prepared using 0.5 μl cDNA reaction as template and run for 30 cycles. 5ul of completed PCR reactions were checked on 1% agarose gels. Target primers were used at 200 nM final concentration and 200 nM actin primers were used in separate control reactions. Four biological replicates were performed for each experiment. The following primers were used for target and control gene amplification:

PLC1 det-fwd2: 5′-GAAGCTGAAGTTCGTCATGG-3′.

PLC1 det-rev2: 5′-CATAGCCACATCCACCATTG-3′.

ACT2 fwd: 5′-AGAAGTCTTGTTCCAGCCCTC-3′.

ACT2 rev: 5′-TTAGAAACATTTTCTGTGAACG-3′.

### T-DNA allele verification

Genomic DNA was prepared from 100 mg of plant tissue using the Qiagen DNeasy plant kit. We used PCR primer sequences provided by SIGnAL primer design tool for genotyping *TTG2* and *AtPLC1* loci towards the identification of a double T-DNA mutant line. Primer sequences are listed below:

LP-TTG2: 5′-TAAAACCAAACGACACCGTTC-3′

RP-TTG2: 5′-TCCAAGTTTGTTGACGATTCC-3′

LP-PLC1: 5′-AAACGCGTTCTCCTTAACCAG-3′

RP-PLC1: 5′-TACTTTGGGTCAACGGTTCTG-3′

LBb1.3: 5′-AAACGCGTTCTCCTTAACCAC-3′.

### Research involving plants

Commonly used and publicly available strains of the laboratory model plant, *Arabidopsis thaliana*, were used in this study. No wild plants or endangered species of plants were used in this study. No field experiments were conducted. Therefore, this study is compliant with relevant institutional, national, and international guidelines and legislation on plant research.

### Supplementary Information

Supplementary Figures.

## Data Availability

Most of the *Arabidopsis* strains used in this study, including the ecotypes Col and L*er* and the insertion mutants *ttg2-3* and *plc1-1D*, are available upon request at the Arabidopsis Biological Resource Center. The transgenic reporter lines and overexpression lines described in this study are avaible free upon request from the Plant Pathways Freshman Research Initiative lab by contacting Dr. Tony Gonzalez. *E. coli* strains carrying promoter:reporter and overexpression constructs are also available upon request from the Plant Pathways lab.
