# Supplementary material for: Genetic interaction between TTG2 and AtPLC1 reveals a role for phosphoinositide signaling in a co-regulated suite of Arabidopsis epidermal pathways
Source: Sci Rep. 2024 Apr 28;14:9752. doi: 10.1038/s41598-024-60530-8 (PMC11056374; doi:10.1038/s41598-024-60530-8)
Supplement: Supplementary file 1 — Supplementary Figures. [file 41598_2024_60530_MOESM1_ESM.pptx]

## Slide 1
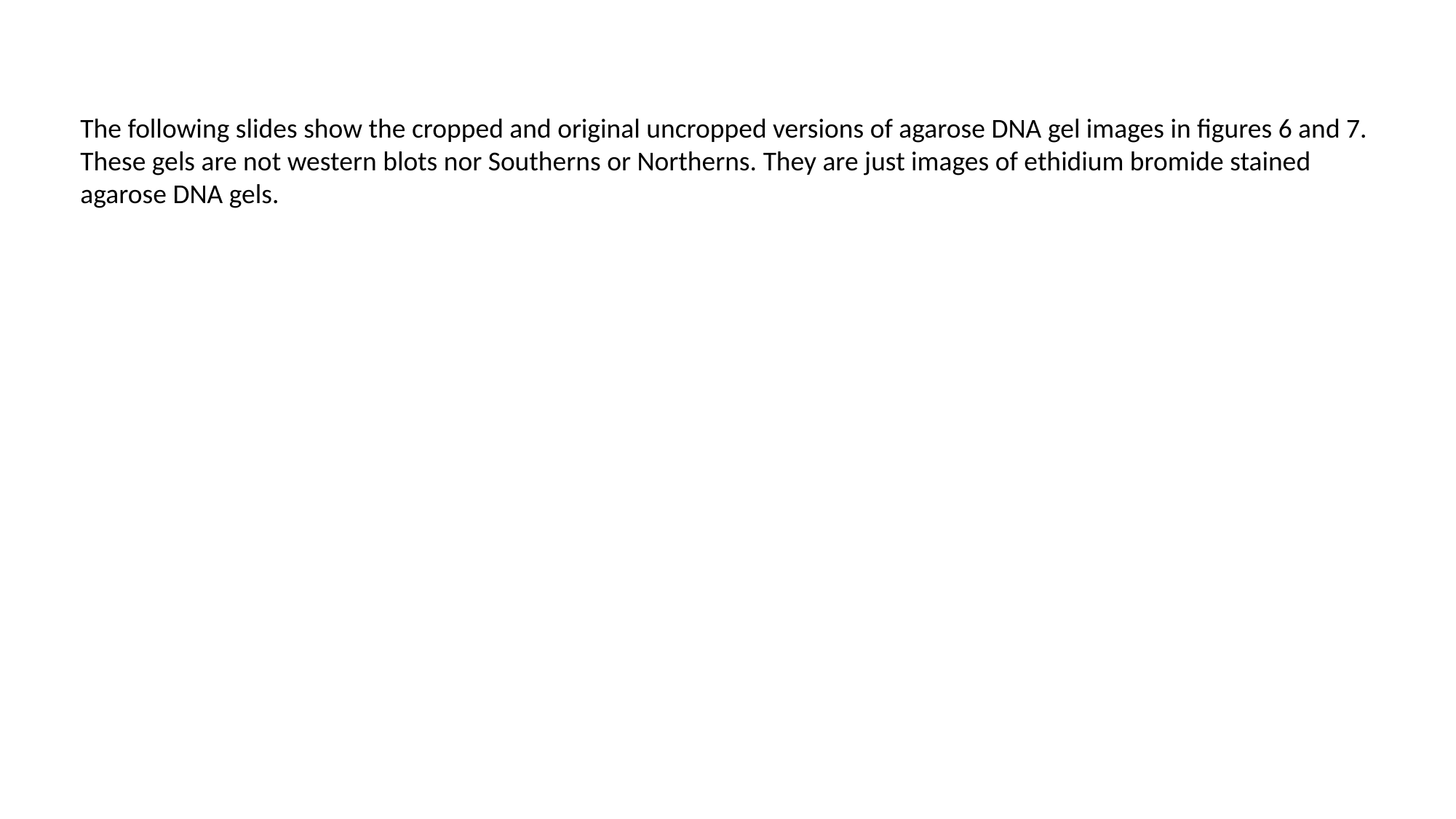

The following slides show the cropped and original uncropped versions of agarose DNA gel images in figures 6 and 7.
These gels are not western blots nor Southerns or Northerns. They are just images of ethidium bromide stained
agarose DNA gels.

## Slide 2
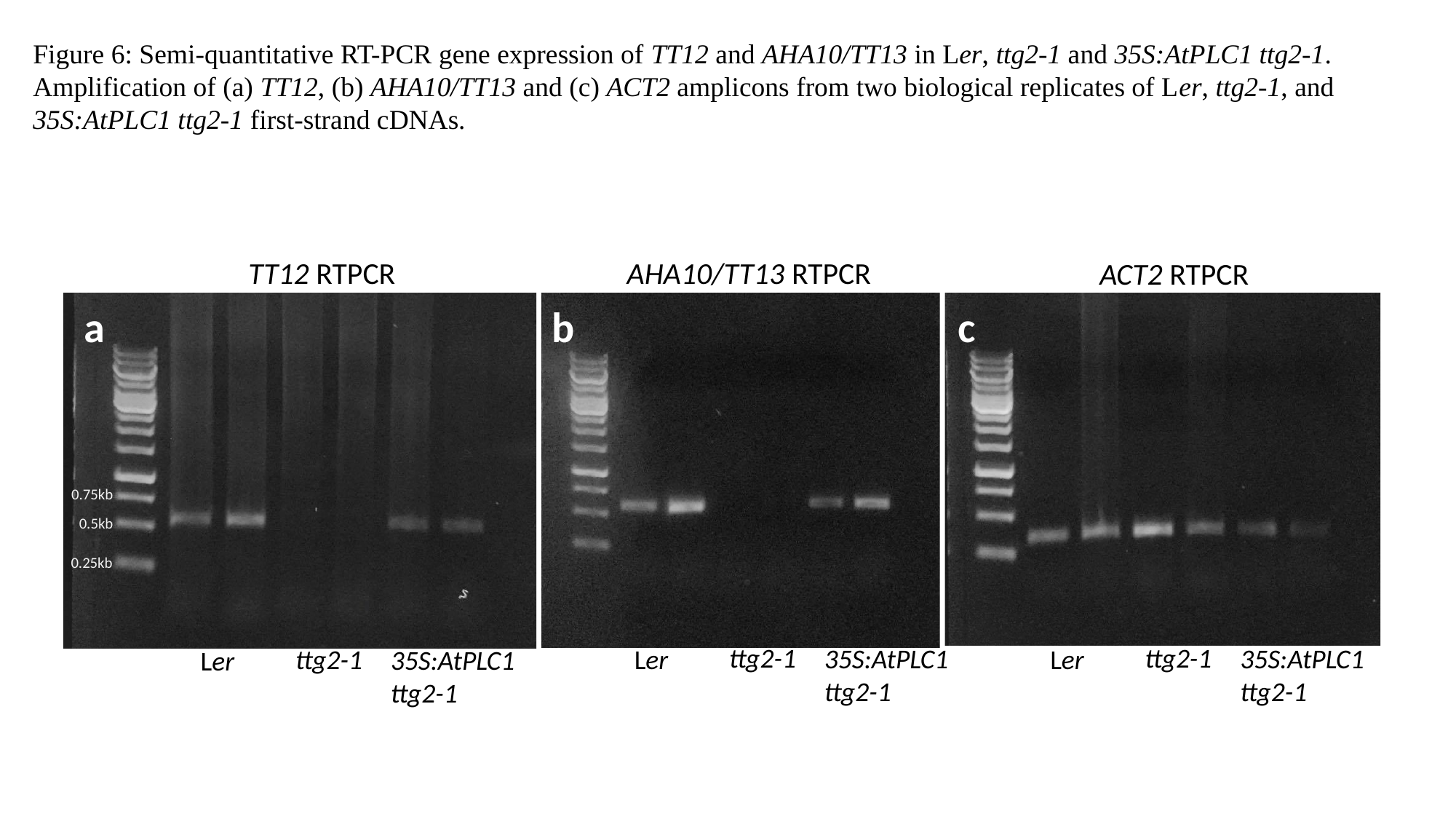

Figure 6: Semi-quantitative RT-PCR gene expression of TT12 and AHA10/TT13 in Ler, ttg2-1 and 35S:AtPLC1 ttg2-1.
Amplification of (a) TT12, (b) AHA10/TT13 and (c) ACT2 amplicons from two biological replicates of Ler, ttg2-1, and
35S:AtPLC1 ttg2-1 first-strand cDNAs.
TT12 RTPCR
AHA10/TT13 RTPCR
ACT2 RTPCR
a
b
c
0.75kb
0.5kb
0.25kb
ttg2-1
ttg2-1
35S:AtPLC1
ttg2-1
35S:AtPLC1
ttg2-1
ttg2-1
Ler
Ler
35S:AtPLC1
ttg2-1
Ler

## Slide 3
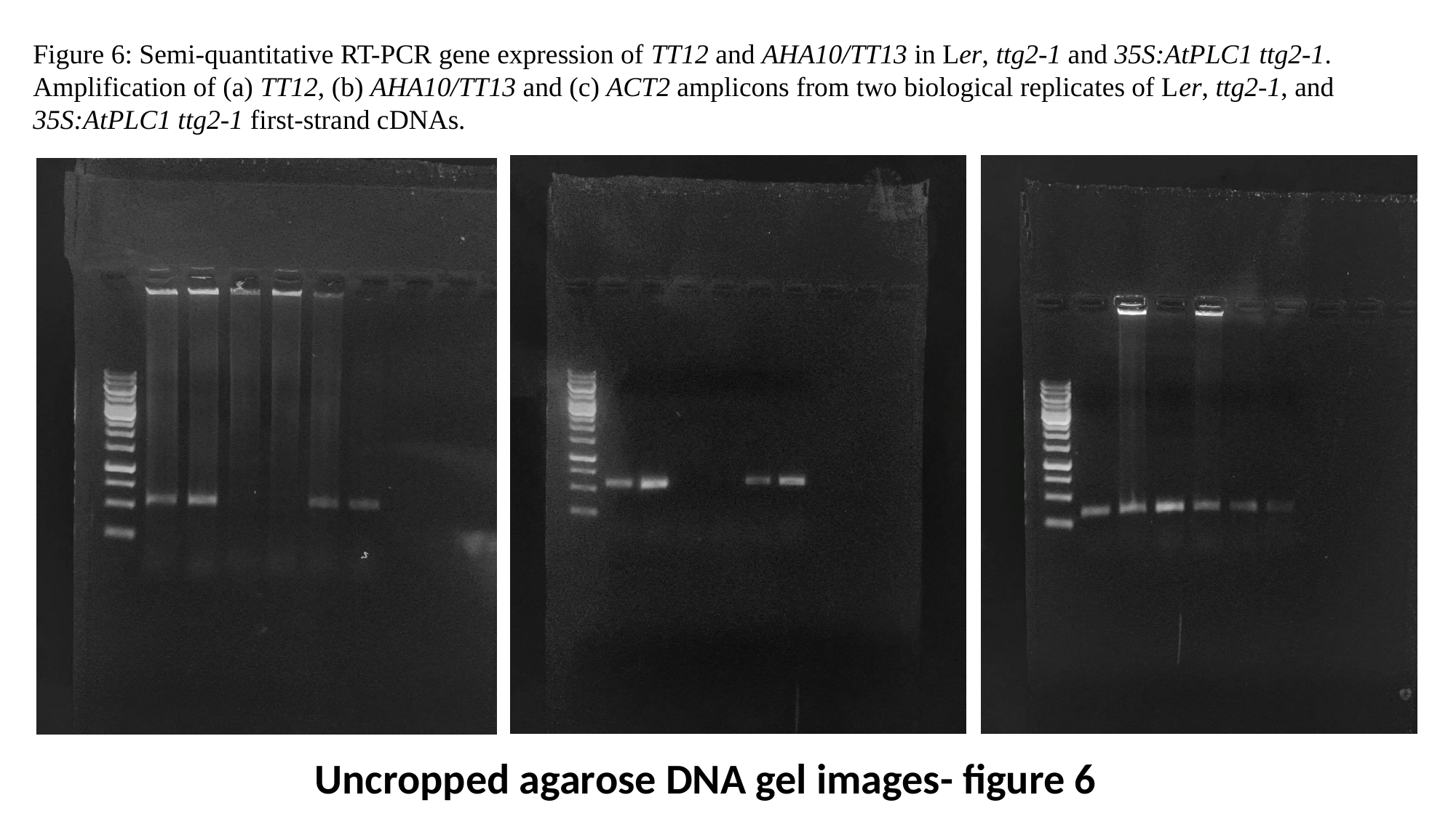

Figure 6: Semi-quantitative RT-PCR gene expression of TT12 and AHA10/TT13 in Ler, ttg2-1 and 35S:AtPLC1 ttg2-1.
Amplification of (a) TT12, (b) AHA10/TT13 and (c) ACT2 amplicons from two biological replicates of Ler, ttg2-1, and
35S:AtPLC1 ttg2-1 first-strand cDNAs.
Uncropped agarose DNA gel images- figure 6

## Slide 4
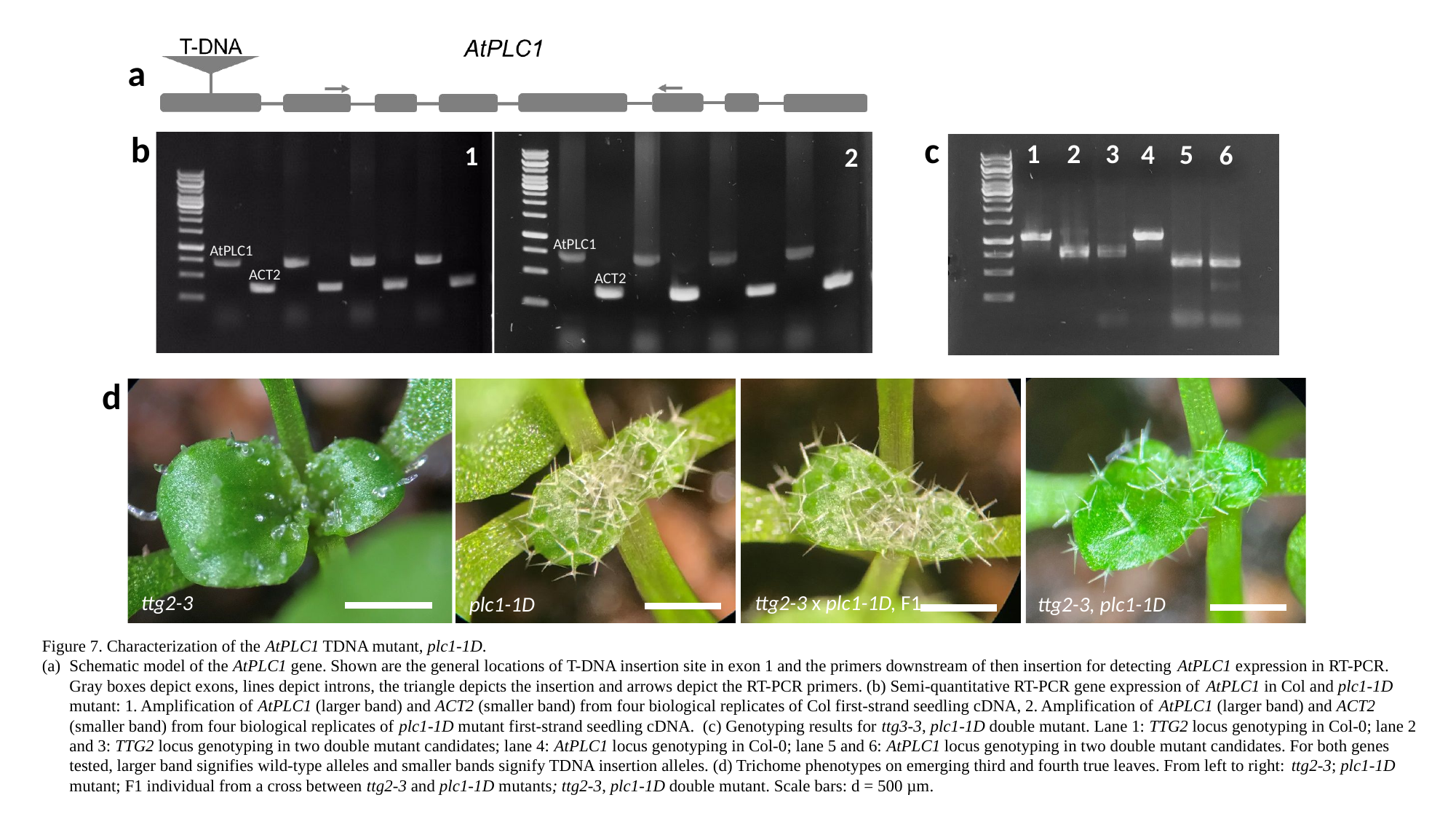

a
b
c
3
2
1
5
4
6
1
2
AtPLC1
AtPLC1
ACT2
ACT2
d
ttg2-3
ttg2-3 x plc1-1D, F1
plc1-1D
ttg2-3, plc1-1D
Figure 7. Characterization of the AtPLC1 TDNA mutant, plc1-1D.
Schematic model of the AtPLC1 gene. Shown are the general locations of T-DNA insertion site in exon 1 and the primers downstream of then insertion for detecting AtPLC1 expression in RT-PCR. Gray boxes depict exons, lines depict introns, the triangle depicts the insertion and arrows depict the RT-PCR primers. (b) Semi-quantitative RT-PCR gene expression of AtPLC1 in Col and plc1-1D mutant: 1. Amplification of AtPLC1 (larger band) and ACT2 (smaller band) from four biological replicates of Col first-strand seedling cDNA, 2. Amplification of AtPLC1 (larger band) and ACT2 (smaller band) from four biological replicates of plc1-1D mutant first-strand seedling cDNA. (c) Genotyping results for ttg3-3, plc1-1D double mutant. Lane 1: TTG2 locus genotyping in Col-0; lane 2 and 3: TTG2 locus genotyping in two double mutant candidates; lane 4: AtPLC1 locus genotyping in Col-0; lane 5 and 6: AtPLC1 locus genotyping in two double mutant candidates. For both genes tested, larger band signifies wild-type alleles and smaller bands signify TDNA insertion alleles. (d) Trichome phenotypes on emerging third and fourth true leaves. From left to right: ttg2-3; plc1-1D mutant; F1 individual from a cross between ttg2-3 and plc1-1D mutants; ttg2-3, plc1-1D double mutant. Scale bars: d = 500 µm.

## Slide 5
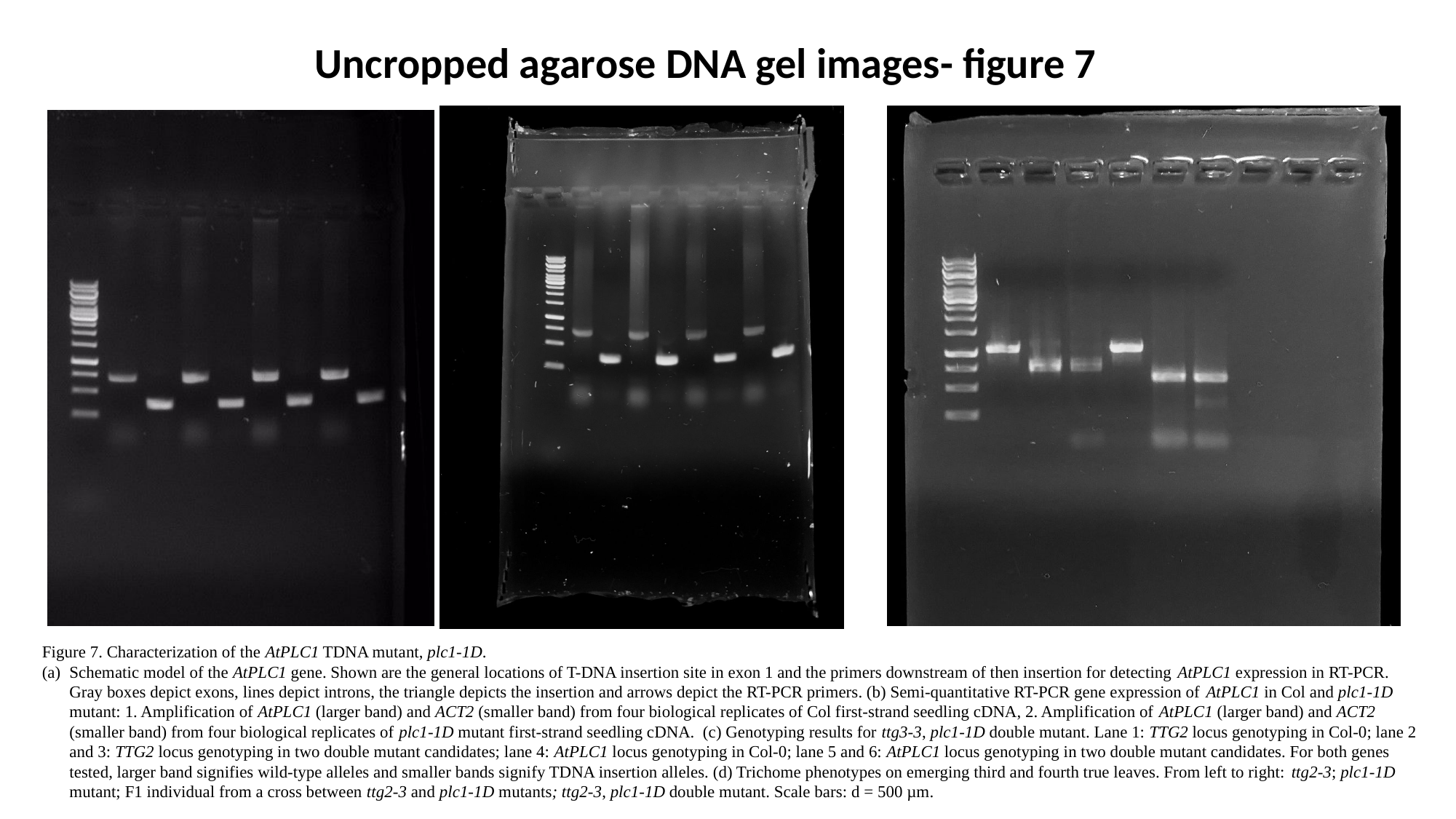

Uncropped agarose DNA gel images- figure 7
Figure 7. Characterization of the AtPLC1 TDNA mutant, plc1-1D.
Schematic model of the AtPLC1 gene. Shown are the general locations of T-DNA insertion site in exon 1 and the primers downstream of then insertion for detecting AtPLC1 expression in RT-PCR. Gray boxes depict exons, lines depict introns, the triangle depicts the insertion and arrows depict the RT-PCR primers. (b) Semi-quantitative RT-PCR gene expression of AtPLC1 in Col and plc1-1D mutant: 1. Amplification of AtPLC1 (larger band) and ACT2 (smaller band) from four biological replicates of Col first-strand seedling cDNA, 2. Amplification of AtPLC1 (larger band) and ACT2 (smaller band) from four biological replicates of plc1-1D mutant first-strand seedling cDNA. (c) Genotyping results for ttg3-3, plc1-1D double mutant. Lane 1: TTG2 locus genotyping in Col-0; lane 2 and 3: TTG2 locus genotyping in two double mutant candidates; lane 4: AtPLC1 locus genotyping in Col-0; lane 5 and 6: AtPLC1 locus genotyping in two double mutant candidates. For both genes tested, larger band signifies wild-type alleles and smaller bands signify TDNA insertion alleles. (d) Trichome phenotypes on emerging third and fourth true leaves. From left to right: ttg2-3; plc1-1D mutant; F1 individual from a cross between ttg2-3 and plc1-1D mutants; ttg2-3, plc1-1D double mutant. Scale bars: d = 500 µm.
